# Development and Preliminary Evaluation of a Teacher-Supervised AI-Assisted Closed-Loop Learning Model for an Undergraduate Animal Physiology Laboratory Course

**DOI:** 10.3390/ani16213379

**Published:** 2026-10-27

**Authors:** Qiongzhen Xian, Shengfeng Chen, Yinshan Bai, Kuiqing Cui, Lian Liu, Wei Wang, Shuo Zhou, Xiaoshu Zhan

**Affiliations:** School of Animal Science and Technology, Foshan University, Foshan 528231, China

**Keywords:** veterinary education, AI-assisted learning, educational technology, blended learning, virtual laboratory

## Abstract

Animal Physiology Laboratory requires students to integrate physiological knowledge, experimental procedures, animal handling, real-time signal interpretation, and post-class reflection. To address the fragmentation of conventional laboratory teaching, this study developed an AI-assisted closed-loop learning model integrating online preparation, virtual simulation, predictive interpretation, hands-on animal experimentation, real-time physiological signal acquisition, troubleshooting-oriented review, and teacher-supervised AI-assisted feedback. Two evaluations were conducted at class-level: (1) a non-concurrent historical cohort comparison of students in Animal Science and Veterinary Medicine who entered the university in 2020 or 2021 (pre-reform cohorts) and 2022 or 2023 (post-reform cohorts); and (2) a cluster-randomized comparison involving 386 Veterinary Medicine students from 14 classes, allocated to either traditional teaching (7 classes; *n* = 190) or the AI-assisted group (7 classes; *n* = 196). The lab course lasted 8 weeks, while the teaching team, instructional content, laboratory resources, assessment criteria, and grading rubrics were matched between groups. The historical cohorts had higher reported final grades after the curriculum reform in both majors (*p* < 0.05). In the cluster randomized comparision, baseline prerequisite-course scores were comparable between the traditional and AI-assisted groups (81.93 ± 0.65 vs. 81.13 ± 0.59, *p* = 0.3807), and the final laboratory scores were higher in the AI-assisted group (94.27 ± 0.27 vs. 89.09 ± 1.16, *p* < 0.01). Although postrequisite-course scores and score gains from prerequisite to postrequisite courses were slightly higher in the AI-assisted group, neither difference was statistically significant (*p* = 0.1470 and *p* = 0.3022, respectively). These findings suggest that the AI-assisted closed-loop learning model can improve course-specific learning performance in veterinary physiology laboratory education. Its longer-term effects on subsequent-course performance require further investigation.

## Simple Summary

Veterinary physiology laboratories require students to connect theory with experimental procedures and physiological responses. This study evaluated a teaching model integrating online preparation, virtual rehearsal, practical experiments, and teacher-supervised feedback. In a class-based comparison, students receiving the model achieved higher laboratory-course scores than those receiving traditional teaching, but subsequent-course performance did not differ significantly. The model may support learning in the laboratory course; its long-term benefits require further study.

## 1. Introduction

The rapid digital transformation of higher education has created new opportunities for reforming veterinary teaching, particularly in laboratory-based courses that require the integration of conceptual understanding, procedural familiarity, practical skills, and analytical reasoning [1]. In recent years, digital teaching platforms, artificial intelligence (AI), virtual simulation, and data-supported learning tools have increasingly been introduced into medical and veterinary education [2,3]. However, the educational value of these technologies does not lie solely in the digitization of individual resources or the deployment of standalone tools. More importantly, they offer the possibility of redesigning the entire learning process in a more coherent, connected, and feedback-rich manner [4,5,6].

Animal Physiology Laboratory is a foundational course for students majoring in veterinary medicine and animal science. It plays an important role in helping students understand physiological mechanisms through direct observation, experimental manipulation, and analysis of real biological responses [7]. Unlike purely theoretical courses, this type of teaching requires students to move continuously between multiple forms of learning: reviewing experimental principles, understanding operational steps, rehearsing procedures, handling experimental animals, recording physiological signals, interpreting images and curves, and reflecting on errors or unexpected outcomes [8]. These demands make the course highly suitable for digital enhancement, but also highly sensitive to weaknesses in instructional design.

In laboratory-based bioscience education, linking pre-laboratory preparation, hands-on work, data interpretation, and post-laboratory reflection can be challenging. Staff surveys have identified student preparedness, confidence, time constraints, and the limited incorporation of inquiry as recurring concerns in undergraduate teaching laboratories [9]. When laboratory activities are predominantly protocol-driven, students may have fewer opportunities to make experimental decisions and engage in scientific reasoning [10]. Pre-laboratory demonstrations have been reported to help students become familiar with equipment and procedures before class, while physiology-education reviews indicate that virtual laboratories can support conceptual learning but may not provide the same experience as hands-on practical work [11,12]. Post-laboratory written work can support the integration of concepts and evidence, but its learning value depends on how the activity is designed and assessed [8,10]. Together, these findings underscore the importance of coordinating preparation, practical activity, interpretation, and feedback, rather than assuming that simply completing a laboratory protocol will connect theory with practice.

Recent developments in smart teaching provide a promising direction for addressing this fragmentation [13]. Online learning platforms can support structured pre-class preparation. Virtual simulation systems can provide repeated rehearsal opportunities before live experimentation [4]. Real-time physiological signal acquisition systems can strengthen the authenticity of experimental observation. In addition, AI-assisted feedback tools can extend post-class formative assessment by supporting scoring, guided questioning, and reflective review [4]. Nevertheless, these resources may still used in parallel rather than as parts of an integrated instructional sequence. A key challenge is therefore not simply whether digital tools are available, but how they are pedagogically organized into a coherent learning process in which preparation informs practical work, experimental observations support interpretation, and subsequent feedback guides reflection and further learning.

We hypothesized that integrating pre-class preparation, virtual rehearsal and interpretation, hands-on experimentation, real-time signal recording, and post-class feedback into a coordinated instructional sequence would better support students’ procedural understanding, predictive reasoning, and interpretation of physiological results than a fragmented sequence of learning activities. To examine this hypothesis, we designed and practiced an AI-assisted closed-loop learning model for the course Animal Physiology Laboratory. Rather than centering the study on the development of a single AI tool, we focused on reconstructing the full instructional sequence by integrating online preparation, virtual simulation, virtual physiology interpretation, hands-on animal experimentation, real-time physiological recording, post-class troubleshooting review, and teacher-supervised AI-assisted feedback. The aim of this study was to explore how a smart-course framework could connect pre-class, in-class, and post-class learning into a continuous instructional loop, thereby improving procedural understanding, predictive reasoning, result interpretation, and formative assessment in veterinary experimental education.

## 2. Course Context and AI-Assisted Closed-Loop Instructional Design

### 2.1. Course Background and Resource Foundation

Animal Physiology Laboratory is a core experimental course serving undergraduate students in veterinary medicine and animal science. The course carries 2 credits and consists of 32 class hours in Foshan University in China. It is closely linked to the theoretical course in animal physiology and is intended to strengthen students’ understanding of physiological regulation, experimental operations, data acquisition, and result interpretation. In recent teaching cycles, the course has been offered to multiple classes of veterinary and animal science students, forming an important component of practical ability training within the curriculum.

**Figure 1:**
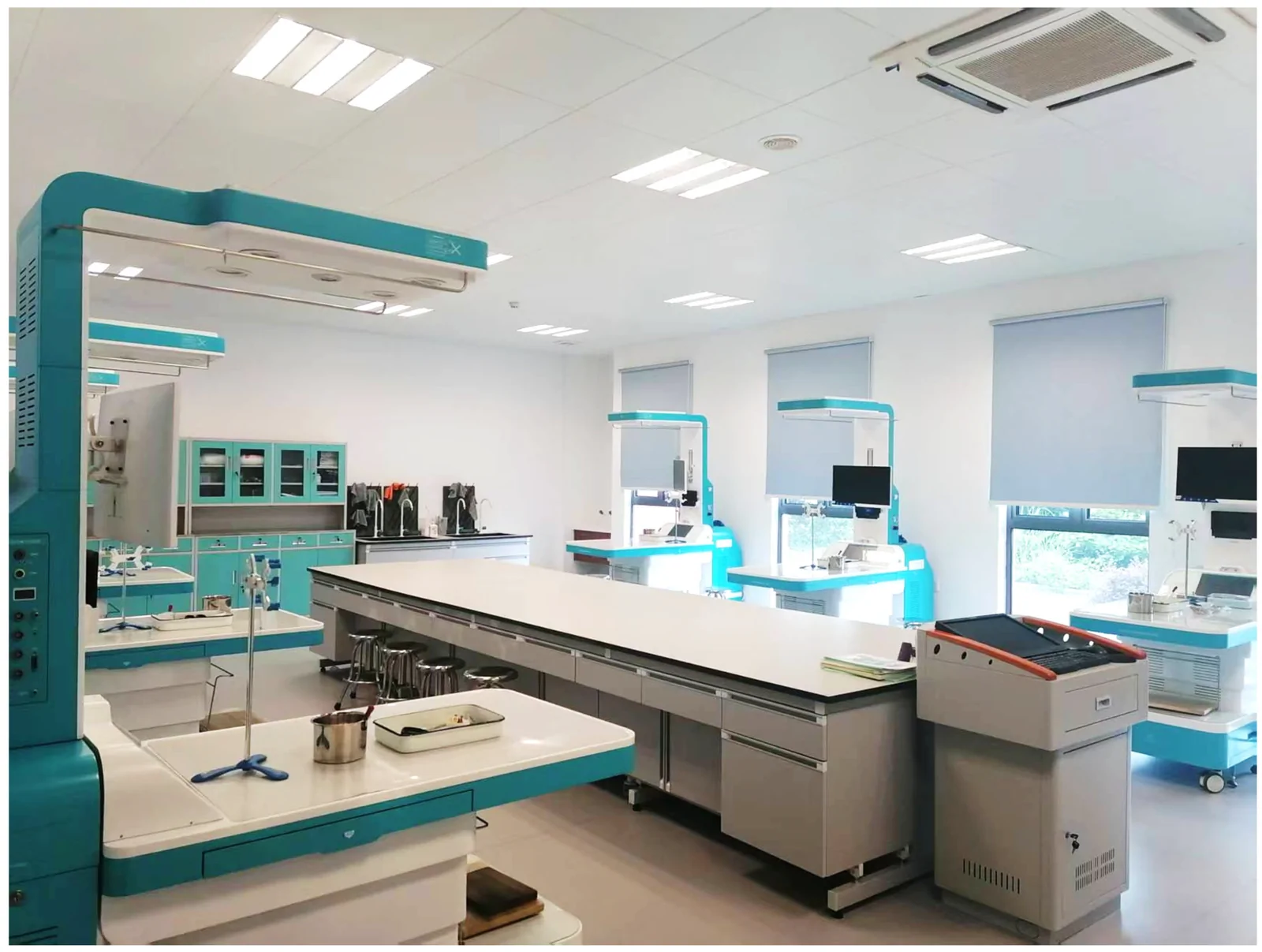
The Animal Physiology Laboratory of Foshan University is equipped with 6 sets of BL-422I information signal acquisition systems.

**Figure 2:**
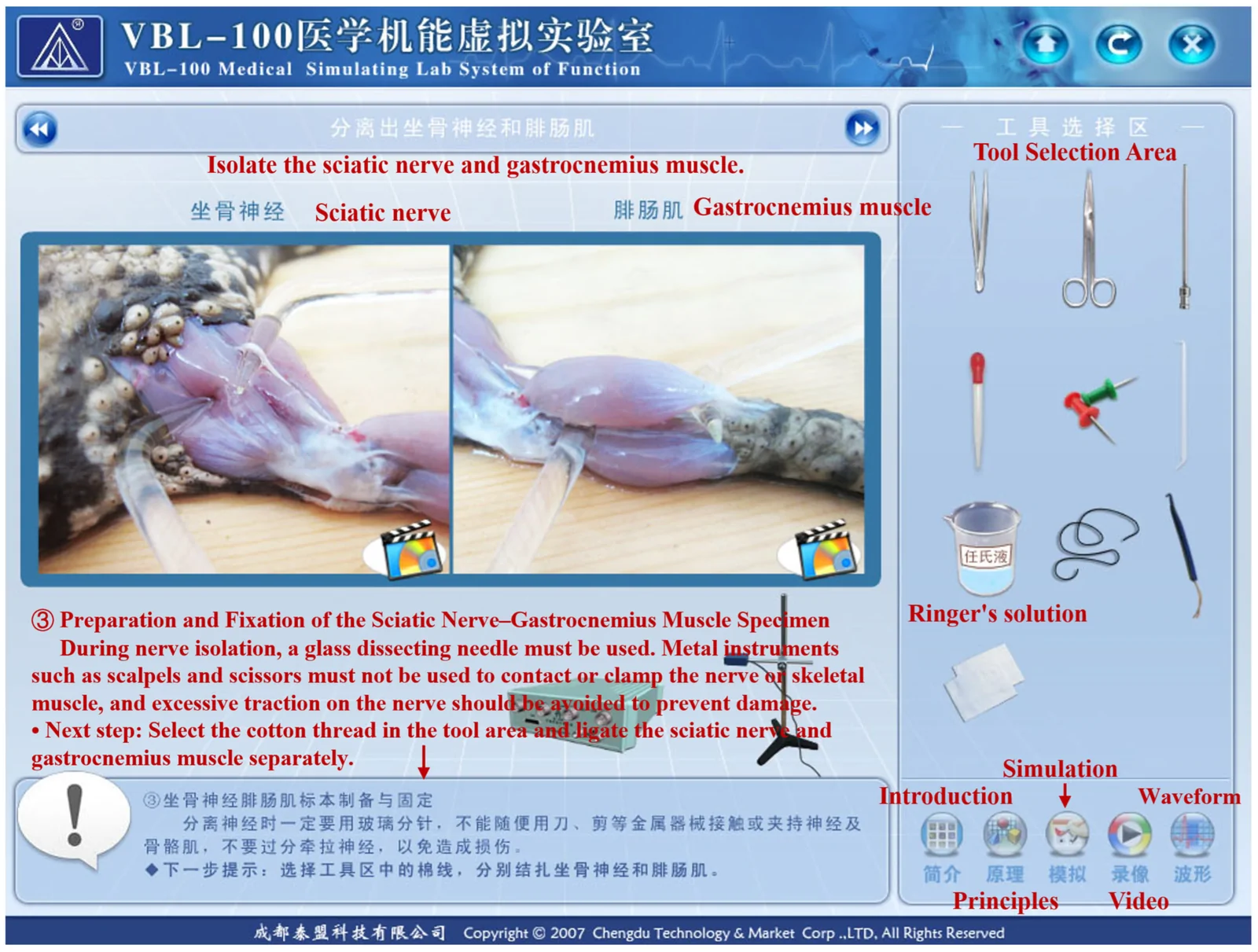
The VBL-100 virtual simulation experiment system. The simulation operation module integrates images, text, and video recordings, resulting in a high degree of realism.

The course had already accumulated a relatively rich resource base before the present instructional redesign. Existing resources included teaching slides, electronic lesson plans, experimental videos, test banks, teaching cases, and virtual simulation materials. A knowledge graph and competency-oriented resource structure had also begun to take shape on the Chaoxing learning platform (https://v8.chaoxing.com/; Version 1.4). In addition, the laboratory had been equipped with six sets of BL-422I intelligent physiological signal acquisition systems (Figure 1, Chengdu Taimeng Software Co., Ltd. Chengdu, China), which enabled the recording and analysis of bioelectrical and non-bioelectrical signals in real time. The teaching team also had access to the VBL-100 virtual simulation system (Figure 2, Chengdu Taimeng Software Co., Ltd.), which provided virtual operation modules relevant to experimental physiology and related disciplines. These existing resources created the foundation for a more integrated smart-course model rather than a fragmented collection of digital materials. Importantly, the present work did not begin with the assumption that a new technology alone could solve all instructional problems. Instead, the course redesign was based on the idea that online resources, simulation tools, laboratory equipment, and AI-assisted feedback should be pedagogically integrated according to the actual logic of student learning. Thus, the major contribution of the current model lies in process-level instructional integration rather than in the isolated deployment of a single digital platform.

### 2.2. Design Rationale for a Closed-Loop Learning Process

The central problem identified in prior teaching practice was the disconnection among different stages of student learning. In many cases, students could complete basic operational steps but lacked a systematic understanding of why each step was necessary, what physiological changes were expected, how signal patterns should be interpreted, and how technical artifacts should be distinguished from genuine biological responses. Such disconnection is particularly problematic in animal physiology experiments, where success depends not only on manual execution but also on the ability to anticipate, observe, interpret, and troubleshoot.

A closed-loop learning process was therefore considered necessary for several reasons. First, students needed stronger structured preparation before entering the laboratory, so that they would begin class with a clearer cognitive map of the experiment. Second, they needed a transition stage between conceptual preparation and live animal operation, in which they could rehearse procedures, visualize the experimental sequence, and develop expectations regarding possible outcomes. Third, hands-on laboratory work needed to remain the central site of authentic skill acquisition, rather than being replaced by simulation. Fourth, post-class review needed to move beyond conventional report writing toward guided reflection on results, errors, and troubleshooting logic. Finally, instructors needed a manageable way to monitor the learning process, provide timely guidance, and review AI-supported feedback without being replaced by it. Based on these needs, we developed an AI-assisted closed-loop learning process that connected the main stages of the course into a continuous instructional chain. The model included pre-class online preparation, in-class virtual rehearsal and predictive interpretation, live animal experimentation and real-time signal acquisition, and post-class result submission with troubleshooting-oriented AI-assisted feedback under teacher supervision. This design was intended to link knowledge acquisition, procedural rehearsal, authentic operation, signal interpretation, reflective consolidation, and formative assessment into one coherent smart-course framework.

### 2.3. Pre-Class Online Preparation

The first stage of the model focused on structured pre-class preparation through the Chaoxing learning platform. Before attending the laboratory session, students were required to review experimental protocols, instructional videos, and knowledge graph materials related to the corresponding experimental topic. The protocols helped students understand the procedural sequence, animal preparation steps, instrumentation requirements, and major operational precautions. The instructional videos offered visual demonstrations of the experimental workflow and key techniques. The knowledge graph provided a structured way for students to connect physiological principles, operational steps, observation targets, and expected signal changes. This stage was designed to transform pre-class learning from passive browsing into an organized process of conceptual and procedural entry. Students were expected not only to know the sequence of operations but also to understand the physiological rationale behind the experiment and to anticipate the possible effects of specific interventions. In this way, pre-class preparation served as the cognitive starting point of the closed-loop model and reduced the likelihood of blind or purely imitative participation during the in-class session.

### 2.4. In-Class Virtual Simulation and Predictive Interpretation

After entering the classroom, students first engaged in virtual rehearsal using the VBL-100 simulation system. This system enabled them to review and practice the overall experimental sequence in a virtual environment before carrying out live animal operations. Through simulation, students were able to repeatedly inspect key steps, familiarize themselves with the procedural order, and reduce uncertainty about the physical experiment. This virtual stage served as a pedagogical bridge between pre-class conceptual preparation and real-world laboratory execution. In addition to procedural rehearsal, students were guided to engage in predictive interpretation within a virtual physiology interpretation environment. In this stage, they were asked to consider what kinds of physiological images, tracings, or signal curves would be expected after specific manipulations and why such changes should occur. This instructional step was particularly important because it moved students beyond “how to do” toward “what to expect” and “how to explain.” As a result, the virtual rehearsal phase was not merely technical simulation; it also functioned as a reasoning-oriented stage in which students linked operational actions to anticipated physiological outcomes.

By embedding predictive interpretation into the in-class virtual phase, the model strengthened the continuity between operation and analysis. Students were not treated as passive executors of protocol but as learners who needed to formulate expectations and explanations before confronting real signals generated in the laboratory.

### 2.5. Group-Based Hands-On Animal Experimentation and Real-Time Physiological Recording

Following the virtual rehearsal stage, students worked in groups to carry out the actual animal experiment. Under instructor guidance, they performed the relevant surgical or experimental procedures using appropriate laboratory animals. This hands-on stage remained indispensable because practical competence in animal functional experimentation requires direct experience with tissue handling, operative coordination, instrumentation, and response to real-time procedural variation. After the animal preparation and intervention steps were completed, the experimental animals were connected to the BL-422I intelligent physiological signal acquisition system. This allowed students to observe and record physiological functions in real time and to obtain corresponding images or curves reflecting the experimental condition. The presence of real-time signal acquisition was crucial to the instructional design because it provided authentic data for interpretation rather than simulated outputs alone. Students could therefore compare what they had predicted during the virtual stage with what was actually recorded during the live experiment.

This real-experiment stage served as the point at which prior knowledge and virtual rehearsal were translated into authentic procedural action and signal-based observation. It also created the basis for subsequent reflective learning, because real experimental results frequently include variation, noise, incomplete responses, or technical issues that are not fully captured in idealized teaching examples. Thus, the live laboratory stage was positioned not as an isolated practical session, but as an integral component in the full learning loop.

### 2.6. Post-Class Submission, Troubleshooting Review, and AI-Assisted Feedback

After the laboratory session, students uploaded their experimental results and related learning outputs to the Chaoxing platform. Post-class learning was designed to continue beyond simple report submission. Students were encouraged to revisit the experimental process, compare their observations with expected outcomes, and consolidate their understanding through a troubleshooting-oriented review environment. This environment emphasized the identification of common errors, interpretation of abnormal or unclear signals, and reflection on possible technical or conceptual causes of discrepancies.

At this stage, an AI-based system was used as an auxiliary feedback component. Students’ open-ended responses were evaluated through AI-assisted scoring and guided follow-up questions. Rather than acting as the central instructional driver, AI functioned as a support tool for extending feedback beyond the limited time available in the classroom. It helped generate immediate prompts, identify missing points in student reasoning, and encourage further reflection. However, teacher supervision remained essential. Instructors reviewed and calibrated AI-supported evaluation results to ensure educational appropriateness, disciplinary accuracy, and consistency with course objectives. This arrangement reflected a deliberate pedagogical stance. The value of AI in this course was not to automate teaching authority, but to expand the possibilities of formative assessment and reflective consolidation while preserving teacher oversight. In other words, AI was embedded into the instructional loop as a supportive element in post-class guided inquiry rather than as a substitute for the instructor.

### 2.7. Teacher Roles in the Closed-Loop Model

The introduction of a smart-course framework did not reduce the importance of the instructor [14]. Instead, it reshaped the teacher’s responsibilities. In the present model, teachers primarily acted as designers of the instructional sequence, organizers of digital and experimental resources, guides during in-class operation, responders to unexpected procedural situations, and reviewers of AI-assisted evaluation. This role transformation is pedagogically significant because it shifts teacher effort from repetitive information delivery toward higher-value tasks such as instructional orchestration, individualized guidance, quality control, and reflective feedback.

Teachers were responsible for preparing and updating experimental protocols, videos, graph-based resources, simulation-linked tasks, and post-class review materials. During the class session, they guided students in translating virtual understanding into live experimental action, monitored the safety and quality of animal procedures, and helped resolve technical difficulties or emergency situations. After class, they examined the appropriateness of AI-supported scoring and questioning, thereby ensuring that AI remained aligned with the educational aims of the course. This teacher-centered but technology-assisted arrangement was fundamental to the feasibility of the model. It ensured that digital tools enhanced, rather than diluted, the professional and pedagogical role of the instructor in veterinary experimental teaching.

### 2.8. Evaluation of Instructional Methods

**Figure 3:**
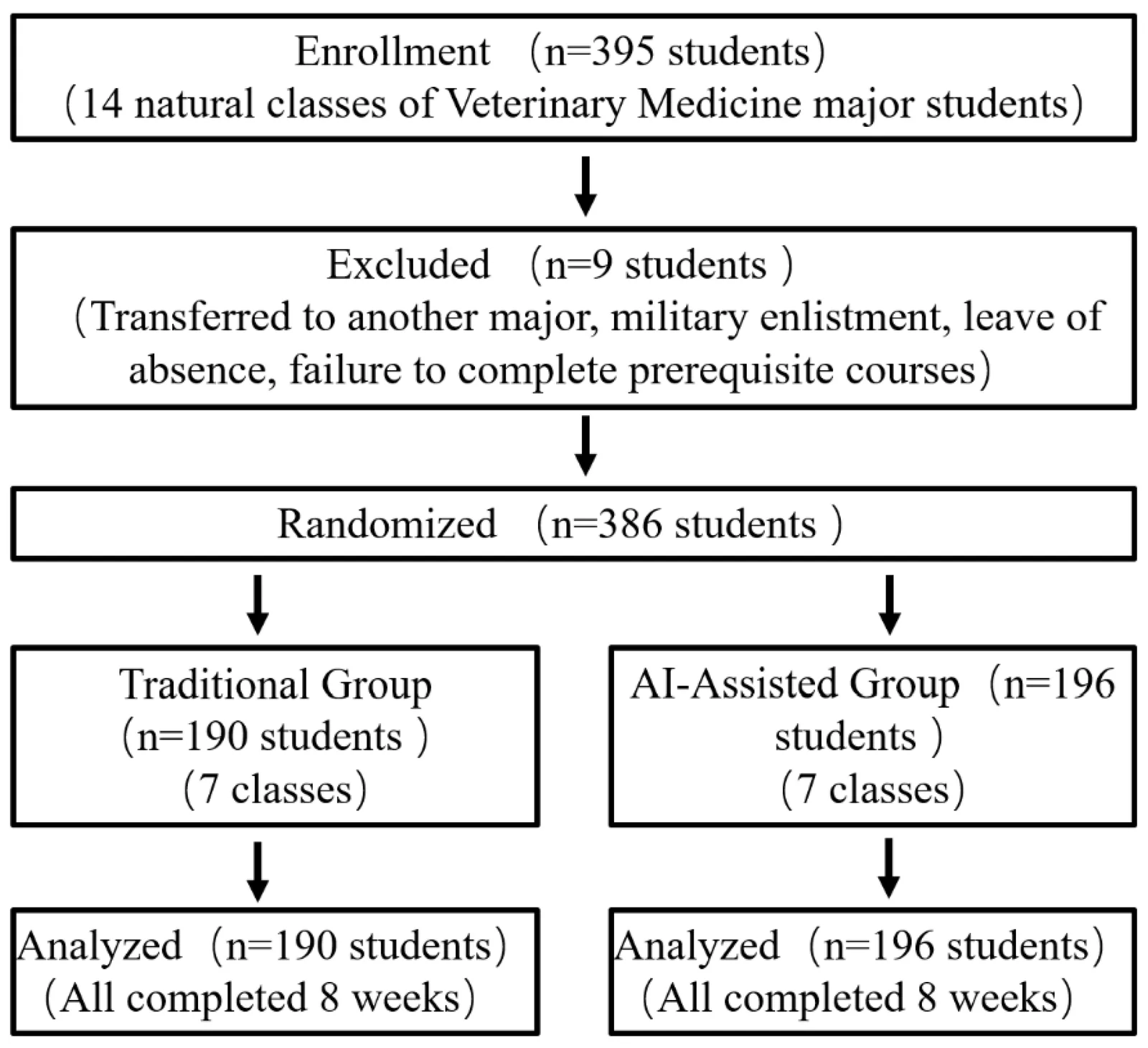
Illustrates the participant flow throughout the trial.

This study comprised two separate assessment approaches. First, historical cohort comparison: final examination scores were compared at the class level between students majoring in Veterinary Medicine and Animal Science who took this course before and after the reform (pre-reform cohorts: undergraduate students who entered the university in 2020 and 2021; post-reform cohorts: undergraduate students who entered the university in 2022 and 2023). For the historical cohort comparison, the unit of analysis was 25 classes, with 11 classes in the 2020-and 2021-entry cohort, and 14 classes in the 2022-and 2023-entry cohort. Results are reported separately for Animal Science and Veterinary Medicine as mean ± SEM. Bars with statistical significance are denoted as ** (*p* < 0.01) using an independent two sample *t*-test. Second, cluster-randomized comparison: 14 intact classes were assigned to traditional teaching group or the AI-assisted teaching group. The sole intervention variable was the use of the AI-assisted closed-loop learning approach in physiology laboratory instruction; all other instructional variables were rigorously controlled to ensure experimental validity. Initially, 395 second-year animal medicine students from 14 intact classes at a college of animal science and technology were recruited. Nine students were subsequently excluded because complete final grades were unavailable due to military enlistment, leave of absence, or other reasons, yielding 386 eligible participants. A cluster randomized controlled design was adopted, with randomization performed at the class level. The 14 classes were allocated to the traditional teaching group (7 classes, 190 students) and the AI-assisted teaching group (7 classes, 196 students) (Figure 3). Independent-samples *t*-tests indicated no statistically significant differences in key baseline variables between the two groups (*p* > 0.05), and no significant differences among classes within either group (all *p* > 0.05). The intervention lasted one semester (8 weeks of physiology laboratory instruction). All statistical analyses were performed at the class level, with each class treated as the unit of analysis. No individual student-level analysis was used to infer intervention effects. Instructional resources were strictly matched between the two groups (identical teaching team, identical theoretical and laboratory content, uniform assessment criteria and grading rubrics), with the teaching model being the only difference. The specific implementation and quality control measures were as follows. The traditional teaching group received the conventional model of “didactic lectures + laboratory instruction + paper-based laboratory reports”; instructors monitored students’ learning progress through in-class questioning and grading of assignments. The AI-assisted group, building on the instruction delivered to the traditional teaching group, additionally incorporated the use of an AI intelligent tutoring system.

## 3. Results

**Figure 4:**
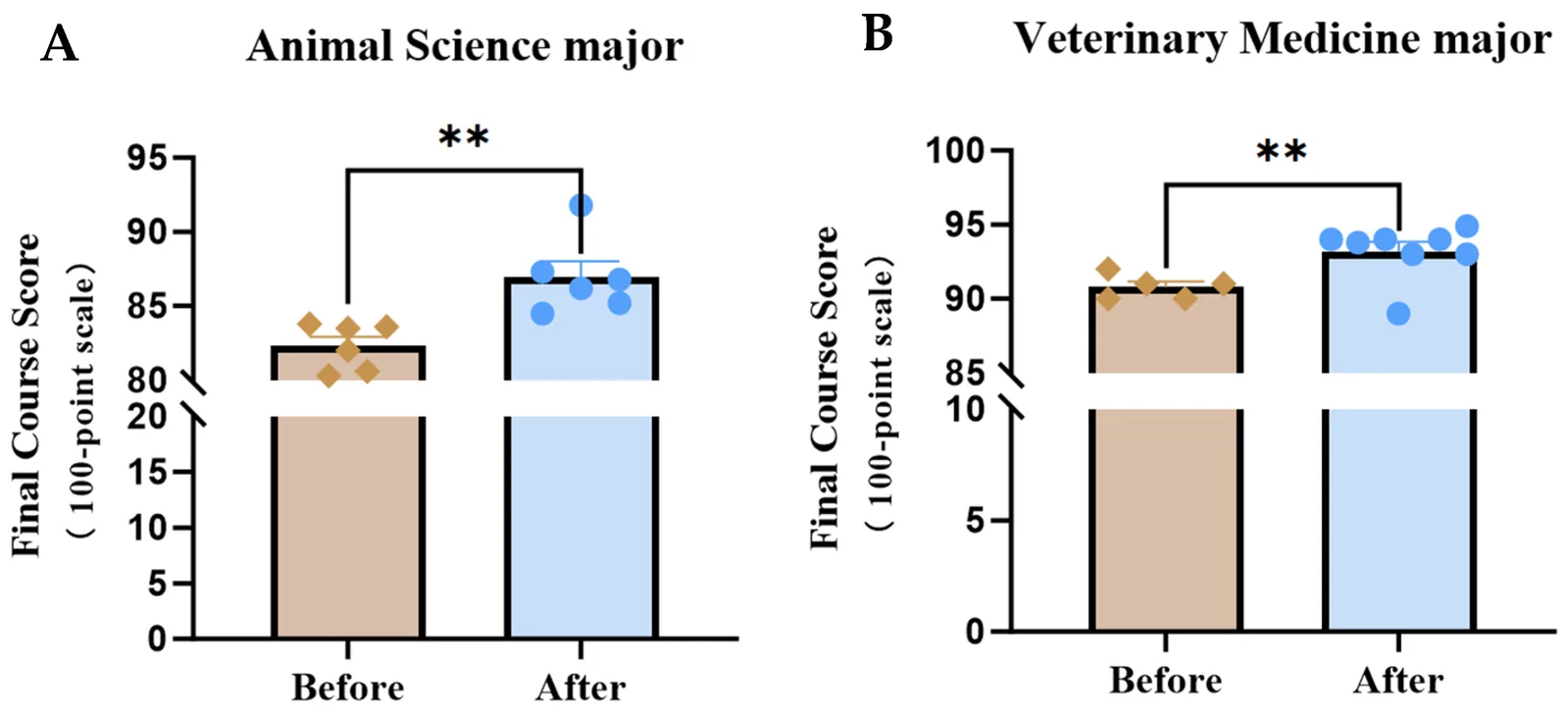
Comparison of student grades in the Animal Physiology Laboratory course before and after curriculum reform. (**A**): Animal Science major; (**B**): Veterinary Medicine major. Results are reported as mean ± SEM. Bars with statistical significance are denoted as ** (*p* < 0.01) using an independent two sample *t*-test.

Historical cohort comparison result showed that, final examination scores of the Animal Physiology Laboratory course at Foshan University were higher in the post-reform cohorts than in the pre-reform cohorts for both the Animal Science major (*p* < 0.05, Figure 4A) and the Veterinary Medicine major (*p* < 0.05, Figure 4B). Results of the cluster-randomized controlled trial are presented in Table 1. The traditional teaching group (*n* = 190) and the AI-assisted group (*n* = 196) had comparable prerequisite-course scores at baseline (81.93 ± 0.65 vs. 81.13 ± 0.59, respectively; *p* = 0.3807). After the 8-week Animal Physiology Laboratory course, the AI-assisted group achieved a significantly higher final examination score than the traditional teaching group (94.27 ± 0.27 vs. 89.09 ± 1.16, respectively; *p* < 0.01). In contrast, The AI-assisted group had slightly higher postrequisite-course scores (85.90 ± 0.62 vs. 84.32 ± 0.77; *p* = 0.1470) and a greater increase from prerequisite to postrequisite-course scores (4.02 ± 0.83 vs. 2.64 ± 0.93; *p* = 0.3022) than the traditional teaching group; however, neither difference was statistically significant. These findings indicate a course-specific advantage of the AI-assisted closed-loop learning approach, whereas a statistically significant advantage in subsequent-course performance was not demonstrated.

## 4. Discussion

The present study explored an AI-assisted closed-loop smart teaching model for Animal Physiology Laboratory education. Its main contribution lies not in the introduction of an isolated digital tool, but in the pedagogical reconstruction of the full learning process. By linking online preparation, virtual rehearsal, live animal experimentation, physiological signal recording, troubleshooting review, and teacher-supervised AI-assisted feedback, the model attempted to address a central weakness of conventional laboratory teaching: fragmentation across learning stages. This design is relevant to veterinary laboratory education, where students need to coordinate procedural skills, theoretical knowledge, observation, and interpretation. A systematic review of simulation-based clinical skills training in veterinary education likewise highlights the importance of aligning learning goals, activities, and outcomes [15]. From this perspective, the value of the present model lies in making the learning trajectory more continuous, visible, and pedagogically manageable.

An important feature of the model is the combined use of virtual and real experimental environments. Virtual simulation was not intended to replace live animal experimentation, nor was it treated as an independent technological showcase. Instead, it served as a rehearsal and transition stage that helped students become more familiar with the sequence of operations and better prepared for live practice. Likewise, the virtual physiology interpretation environment did not replace authentic data interpretation but prepared students to think predictively before observing real recorded signals. This suggests that digital learning environments may be most valuable when they function as bridges between conceptual preparation and authentic experimentation rather than as substitutes for laboratory experience [16]. A systematic review indicates that virtual physiology laboratories effectively support conceptual understanding, although their effects on motivation and skill acquisition remain inconclusive; blended virtual laboratories are at least as effective as in-person laboratories for conceptual learning [12]. Another important issue concerns the role of AI in the course. In many discussions of smart teaching, AI is presented as a transformative driver [17]. However, in the present study, such a framing would not be pedagogically appropriate or empirically justified. The current course model used AI in a more limited but realistic way, primarily for post-class scoring support and guided questioning. This auxiliary role is important for two reasons. First, it allows AI to extend the timeliness and reach of formative feedback without overstating its authority. Second, it preserves teacher responsibility for reviewing disciplinary accuracy, educational appropriateness, and fairness in assessment. In this sense, the model supports an “AI-assisted rather than AI-dominated” approach to veterinary education. AI-assisted feedback may have extended opportunities for post-class review by providing students with prompts and feedback on their written responses. Research on educational feedback indicates that its effects vary substantially according to the information provided and the learning outcome being assessed [18]. A systematic review of generative AI for automated feedback in higher education identifies potential uses while emphasizing the importance of integrating these systems with human instruction [19]. A randomized study in medical education also reported improved clinical decision-making scores when structured LLM-generated feedback was added to simulation practice, although its small sample and different learning context limit direct comparison with the present course [20]. These studies provide a rationale for further evaluating AI-assisted feedback, but they do not establish that AI itself produced the grade differences observed here. In addition, a survey of veterinary students reported both perceived relevance of ChatGPT (version 3.5) and concerns about its accuracy; such perception data provide disciplinary context, not evidence of improved learning outcomes [21].

The study also highlights a meaningful shift in teacher roles within smart-course construction. Teachers are not displaced by digital technologies; rather, their work becomes more focused on instructional design, resource integration, in-class guidance, troubleshooting, and quality control. This redistribution of responsibility may improve the overall efficiency and educational value of teaching, provided that adequate support and training are available for instructors. In laboratory courses, where unpredictable situations and procedural variation are common, teacher presence remains indispensable.

Our study reported higher final laboratory examination scores in the post-reform cohorts than in the pre-reform cohorts for both majors. In the class-level randomized comparison, students in the AI-assisted group also had higher final laboratory scores than those receiving traditional teaching. However, scores in the subsequent course and gains from prerequisite to subsequent-course assessments did not differ significantly between groups. Taken together, these results suggest a possible benefit of the instructional package for performance in the target laboratory course, but do not establish broader or sustained gains in subsequent learning. The historical comparison is particularly difficult to interpret causally because the cohorts were non-concurrent and may have differed in composition, pandemic-related learning conditions, or course delivery. Moreover, the randomized comparison tested a multi-component package, so the contribution of AI cannot be separated from the effects of simulation, structured preparation, feedback, or other elements of the redesign.

Several limitations should be acknowledged. First, the present study mainly reports the design logic and preliminary practice of the model rather than large-scale quantitative validation of learning outcomes. Future studies should incorporate more systematic evidence, such as student performance comparisons, questionnaire-based learning experience evaluation, and analysis of common error patterns. Second, although AI-assisted feedback was integrated into the post-class stage, its scoring consistency and guidance quality still require continuous calibration and review. Future research should prospectively archive paired AI-generated and instructor-finalized scores and quantify agreement using metrics such as Cohen’s kappa or the intraclass correlation coefficient. Nevertheless, because the intervention combined AI feedback with other instructional elements, the final course score difference cannot be attributed to AI alone. Third, the current implementation focused on representative modules within the course and can be further expanded to include a wider range of experimental themes and deeper data analytics. Despite these limitations, the present work provides a feasible framework that can be iteratively refined and adapted to other laboratory-based teaching contexts in veterinary education.

## 5. Conclusions

This study implemented an AI-assisted closed-loop learning process for an undergraduate Animal Physiology Laboratory course. The findings suggest that the integrated instructional package may support student performance in the target laboratory course; however, because the intervention combined multiple components, the contribution of AI-assisted feedback cannot be isolated. The model’s main contribution is to provide a structured approach for connecting preparation, rehearsal, authentic experimentation, interpretation, and feedback within a continuous learning process. Future studies should evaluate the model across a broader range of course modules and assess learning outcomes, feedback quality, and agreement between AI-generated and teacher-confirmed assessments. Once these components have been adequately validated, multimodal AI agents and learning analytics could be explored to integrate students’ written responses, experimental data, and learning-process information, and to provide more targeted feedback under teacher supervision. The applicability of these extensions will require further empirical evaluation.

## Figures and Tables

**Table 1:** Comparison of prerequisite course scores, Animal Physiology Laboratory course final score, Postrequisite Course score and Postrequisite vs. Prerequisite Course Score Difference between Traditional teaching group and AI-Assisted Group.

Assessment	Traditional Group	AI-Assisted Group	Minimum (T vs. AI)	Maximum (T vs. AI)	*p* Value
Prerequisite course score (baseline)	81.93 ± 0.65	81.13 ± 0.59	79.4, 78.9	84.4, 82.7	0.3807
Animal Physiology Laboratory course final score	89.09 ± 1.16	94.27 ± 0.27	86.00, 92.90	93.70, 94.90	<0.01
Postrequisite Course score	84.32 ± 0.77	85.90 ± 0.62	82.60, 84.00	86.80, 87.20	0.1470
Postrequisite vs. Prerequisite Course Score Difference	2.64 ± 0.93	4.02 ± 0.83	0.70, 1.70	5.90, 6.00	0.3022

Notes: Data are presented as mean ± Standard Error of the Mean. Statistical significance are analyzed using an independent two sample *t*-test. T vs. AI: Traditional teaching group vs. AI-Assisted group. Prerequisite courses include Organic Chemistry, Analytical Chemistry, Animal Anatomy, Zoology, Animal Biochemistry, Animal Histology and Embryology, and their corresponding laboratory courses. Postrequisite courses include Veterinary Pharmacology, Animal Pathological Anatomy, Veterinary Clinical Diagnostics, and Traditional Chinese Veterinary Medicine, along with their corresponding laboratory courses.

## Data Availability

All data supporting the findings of this study are included within the article. No additional datasets were generated.
